# Cooccurrence of Talon's Cusp with Dens Invaginatus in the Maxillary Lateral Incisor: A Case Report with Review of Literature

**DOI:** 10.1155/2022/9165574

**Published:** 2022-02-22

**Authors:** Snehashish Ghosh, Safal Dhungel, Bhawana Subedi, Subi Pradhan

**Affiliations:** ^1^Department of Oral Pathology, College of Medical Sciences, Bharatpur, Nepal; ^2^Department of Oral and Maxillofacial Surgery, College of Medical Sciences, Bharatpur, Nepal; ^3^Department of Oral Medicine and Radiology, College of Medical Sciences, Bharatpur, Nepal; ^4^College of Medical Sciences, Bharatpur, Nepal

## Abstract

Morphogenic developmental anomalies are common in maxillary lateral incisors, but simultaneous occurrence of two developmental anomalies in a single tooth is relatively uncommon. In this case report, we present a case of cooccurrence of the talon's cusp with dens invaginatus in the left lateral incisor tooth. Early diagnosis and prompt treatment of such cases are important to prevent any untoward consequences.

## 1. Introduction

Talon's cusp is an additional entity, projecting from the lingual surface/cingulum area of a maxillary or mandibular anterior tooth. It is an anomalous structure resembling an eagle's talon, so the name talon's cusp has evolved. It is composed of coronal hard and soft tissue structures similar to a normal tooth. It blends smoothly with the tooth except there is a deep developmental groove where the cusp blends with the sloping lingual tooth surface [1]. The first case of the talon's cusp was identified by Mitchell [2].

Sicher and Bhaskar [3] proposed that a disturbance in the morphodifferentiation could lead to the development of the talon's cusp, whereas Hattab et al. [4] thought that the talon's cusp might be a result of outward folding of the inner enamel epithelium with concurrent focal hyperplasia of the dental papilla. It could be one of the presenting features of Mohr syndrome, Rubinstein-Taybi syndrome, and Sturge-Weber syndrome [5–8]. Some authors consider talon's cusp as a hamartoma also [9].

Various clinical problems like attrition, compromised aesthetics, displacement of the involved tooth, interference of tongue space, increased susceptibility to dental caries, and occlusal disharmony could be associated with the talon's cusp [10, 11].

Dens invaginatus/dens in dente is a developmental anomaly that arises as a result of infolding in the surface of the crown/root. Increased localized external pressure, focal growth retardation, and focal growth stimulation in certain areas of the tooth bud are the possible causes of this condition. Siraci et al. and Lwin et al. contemplated an initial contortion followed by the subsequent protrusion of the fragment of the enamel organ could lead to the formation of enamel-lined infolding at the cingulum area even at the incisal tip [10, 12].

It is seen in the order in maxillary lateral incisors, central incisors, canine, premolar, and molar teeth. Usually, it is unilateral, but when occurring in the maxillary central, it could be bilateral [12]. An analogous form of invagination occasionally occurs in the roots of teeth [10]. It results from an infolding of Hertwig epithelial root sheath and takes its origin within the root after development is complete. There is no gender predilection for this condition [12, 13].

Radiographically, the involved tooth may depict an infolding of enamel and dentin reaching towards the pulp cavity or even rarely extending to the root apex. Histopathologically, hypomineralized enamel is noted at the site of invagination, making that site vulnerable for the development of dental caries and penetration of microorganisms deep into the pulpal area leading to pulpal necrosis and initiation of periapical inflammatory processes [10, 12, 14].

Here in this article, we report a rare case of cooccurrence of the talon's cusp with dens invaginatus in the maxillary lateral incisor.

## 2. Case Report

A 25-year-old female patient reported to the dental outpatient department of the College of Medical Sciences, Bharatpur, Nepal, with the chief complaint of the fractured tooth in the upper tooth region since 9 yrs. On extraoral examination, the patient had a prognathic mandible with an asymmetric smile, and the midline was shifted towards the right by 2 mm.

**Figure 1:**
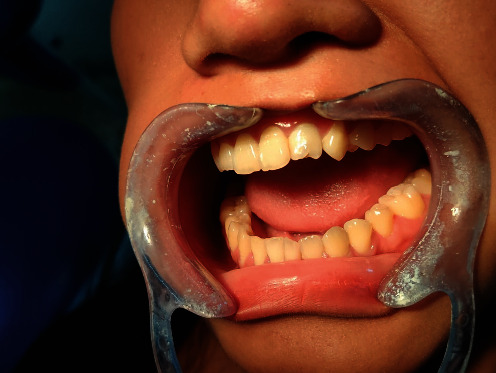
Full mouth intraoral image of the patient.

**Figure 2:**
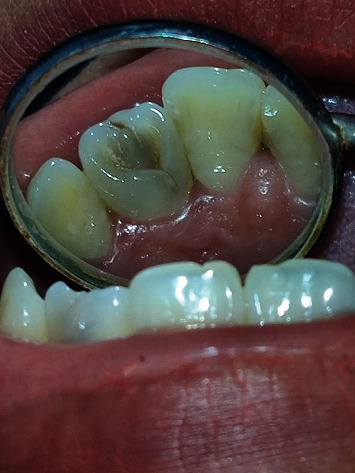
Clinical picture showing talon's cusp with dens invaginatus in maxillary left lateral incisor.

Intraoral examination revealed the patient was in the permanent dentition stage with occlusal caries in the upper right back posterior region. She had a class I occlusal relationship. The full mouth intraoral picture is presented (Figure 1). A prominent tubercle in the lingual aspect of the upper left lateral incisor extends from the cingulum area to the incisal edge (Figure 2). This was diagnosed as a talon's cusp. The tooth was discolored, and a crack line was present on the palatal aspect. The patient did not have any problems regarding speech, mastication, and occlusion. The tooth responded well during the cold test and electric pulp test. On both horizontal and vertical percussion, the involved tooth did not show any pain.

**Figure 3:**
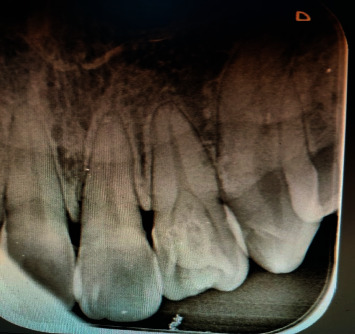
Intraoral periapical radiograph showing talon's cusp with dens invaginatus in maxillary left lateral incisor.

On radiographic evaluation, a shallow V-shaped, tapered radio-opaque structure extending from the cervical third of the crown was noted. A dens invaginatus extending apically beyond the cementoenamel junction was also noted in the same tooth (Figure 3). The apical closure of the tooth was completed. No sign of periapical pathology was witnessed.

According to clinical and radiographic findings, the case was confirmed to be type II talon's cusp together with type I dens invaginatus. As the anomaly enhances plaque accumulation and makes the tooth vulnerable to dental caries, it was advised to fill the developmental groove with sealant for preventive purposes.

As the condition was asymptomatic, the patient did not want to get any treatment done for the involved tooth. The patient was advised to follow the routine brushing practice and maintain optimum oral hygiene and seek regular dental checkups. The written consent was taken from the patient for the publication of the clinical photographs and the X-ray record.

## 3. Discussion

Anomalies like talon's cusp, dens invaginatus, and palatogingival groove are fairly seen in lateral incisor, although the exact cause for this remains unknown [15–17]. However, the simultaneous occurrence of two or more anomalies in a single tooth is relatively rare, and here, in this case, we reported the concurrent occurrence of a talon's cusp with dens invaginatus in the maxillary left lateral incisor of the same patient. Talon's cusp reported, in this case, is a “semitalon,” according to the classification proposed by Hattab et al. [4], which is mentioned in Table 1.

Studies have shown that the prevalence of talon's cusp varies in different ethnic groups ranging from 0.06% in Mexican children to about 7.7% in Indian children. Mongolians show a higher incidence rate of talon's cusp when compared with Caucasians and Negroes [15–18]. Talon's cusp can occur concurrently with other anomalies like bifid cingulum, dens invaginatus, impacted mesiodens, macrodontia, odontoma, and supernumerary tooth [15, 19–21]. These anomalies can alter the vitality of the pulp and can remain asymptomatic [10, 15] as well and get diagnosed under routine radiographic examination [10, 12, 15] which happened in the present case.

Dens invaginatus carries a significantly increased risk of developing into a pulpal pathology. The invaginated portion communicates with the oral cavity, facilitating the ingress of irritants and microbes into the pulp causing pulpal and periapical pathosis [15, 22]. Its incidence is 0.04 to 10% and is usually detected on a routine roentogenic evaluation [23]. As these pathologies are developmental, the apical root-end closure is questionable if there is pulpal involvement in such a tooth with an open apex [15]. Dens invaginatus reported in this case belongs to the type II variant as per Oehlers classification of dens invaginatus [24], which is mentioned in Table 2. Dens invaginatus might coexist with other developmental anomalies like double teeth, shoveling of the incisor, and multirooted anterior teeth [15, 16, 25, 26]. The documented cases, presenting with the cooccurrence of talon's cusp with dens invaginatus, are summarized in Table 3.

The treatment approach varies from case to case. An asymptomatic talon's cusp without any occlusal interference is left untreated, and vigorous oral hygiene and routine monitoring are good enough, whereas any interference with speech and occlusion requires reduction of the cusp with the application of a desensitizing agent; sometimes intentional endodontic treatment and coverage by full-thickness crown may be required to prevent dental caries and premature tooth loss. The management of dens invaginatus may range from simple oral hygiene instructions, frequent monitoring, and conservative restoration to endodontic treatment, which depends exclusively on the presenting symptoms [10, 12, 15].

The clinical significance of talon's cusp and dens invaginatus is they could be associated with compromised function and esthetics, and as they serve an attachment area for the attachment of food particles, dental plaque, they have a high chance to get affected by the carious process [12, 15]. The message to the clinicians through this case report is that whenever such a case is encountered, early diagnosis and prompt treatment should be performed to restore the function, improve the prognosis, minimize potential complications, and improve the quality of life of the patient.

Based on the research data, both talon's cusp and dens invaginatus are seen unilaterally on the lateral incisors [12]. Although both of these developmental anomalies are not uncommon, based on the knowledge of the authors, the concurrent occurrence of talon's cusp with dens invaginatus in a single tooth has not been reported in the Nepalese population till date.

## 4. Conclusion

To summarize, talon's cusp and dens invaginatus are the developmental anomalies noted in the anterior teeth. They could be asymptomatic or could be associated with the symptoms mentioned in this case report. Its early detection and prompt intervention can alleviate the symptoms in symptomatic cases, prevent premature tooth loss, enhance esthetics, and overall improve the well-being of the patient.

## Figures and Tables

**Table 1:** Classification of talon's cusp proposed by Hattab et al. [4].

Types	Description
Type I	True talon—distinctive additional cusp projecting from the lingual/cingulum surface of maxillary/mandibular anterior teeth to at least half a distance from the amelocemental junction to the incisal edge
Type II	Semitalon—an additional cusp of 1 mm or more that extends less than half of the distance from the amelocemental junction to the incisal edge, and it blends well with the lingual surface of the teeth.
Type III	Trace talon—prominence/variation in the cingulum, could be conical, bifid, or tubercle-like

**Table 2:** Classification of dens invaginatus proposed by Oehlers et al. [24].

Types	Description
Type I	Invagination is restricted to the crown up to the cementoenamel junction.
Type II	Invagination extends into the root beyond the cementoenamel junction.
Type IIIa	Invagination extends into the tooth with lateral communication to the periodontal ligament.
Type IIIb	Invagination extends into the tooth with apical communication to the periodontal ligament.

**Table 3:** Summary of the reported cases of talon's cusp/dens evaginatus with dens invaginatus documented till date.

Serial No.	Author/year	Sex	Primary anomaly	Tooth involved	Associated anomaly	Treatment performed
01	Lorena et al./2003 [27]	Male	Talon's cusp	Maxillary left lateral incisor	Dens invaginatus	No treatment

02	Tiku et al./2004 [18]	Female	Talon's cusp	Maxillary left lateral incisor	Dens invaginatus	No treatment

03	Mupparapu et al./2004 [28]	Male	Dens evaginatus	Maxillary right lateral incisor	Dens invaginatus	Prophylactic treatment with glass ionomer cement

04	Siraci et al./2008 [10]	Female	Talon's cusp	Mandibular right central incisor	Dens invaginatus	Grinding followed by prophylactic sealing with flowable composite

05	Anthonappa et al./2008 [29]	Female	Dens evaginatus	Maxillary right lateral incisor	Dens invaginatus	Prophylactic treatment with glass ionomer cement

06	Vardhan et al./2010 [30]	Male	Talon's cusp	Maxillary right and left central and lateral incisor	Dens invaginatus	No treatment

07	Sarraf-Shirazi et al./2010 [31]	Male	Talon's cusp	Maxillary right and left central incisor	Dens invaginatus	Endodontic treatment

08	Yadav et al./2011 [15]		Talon's cusp	Maxillary left lateral incisor	Dens invaginatus	Enameloplasty with prophylactic composite resin treatment

09	Nagaveni et al./2011 [32]	Male	Talon's cusp	Mandibular left central incisor	Dens invaginatus	Prophylactic treatment with glass ionomer cement

10	Akers et al./2014 [33]	Male	Dens invaginatus	Maxillary left lateral incisor	Dens evaginatus	Endodontic treatment

11	Kasat et al./2014 [34]	Female	Talon's cusp	Maxillary left lateral incisor	Dens invaginatus, radicular cyst	Endodontic treatment of the teeth with enucleation of the cyst

12	Babaji /2015 [35]	Male	Dens invaginatus	Supplemental maxillary left central incisor	Dens evaginatus	Extraction as both talon's cusp and dens invaginatus were there on supplemental tooth

13	Lwin et al./2017 [12]	Male	Talon's cusp	Maxillary right lateral incisor	Double dens invaginatus	Restoration with pit and fissure sealant

14	Present case	Female	Talon's cusp	Maxillary left lateral incisor	Dens evaginatus	No treatment

## Data Availability

The figures/data have already been made available in the manuscript.
